# Is Household Wealth Associated With Use of Long-Acting Reversible and Permanent Methods of Contraception? A Multi-Country Analysis

**DOI:** 10.9745/GHSP-D-15-00234

**Published:** 2016-03-25

**Authors:** Jorge I Ugaz, Minki Chatterji, James N Gribble, Kathryn Banke

**Affiliations:** a Abt Associates Inc, Strengthening Health Outcomes through the Private Sector (SHOPS) Project, Bethesda, MD, USA Now with Mathematica Policy Research, Washington, DC, USA; b Abt Associates Inc, SHOPS Project, Bethesda, MD, USA; c The Palladium Group, Washington, DC, USA

## Abstract

As programs continue to expand access to family planning information, services, and products, it is critical that these efforts be undertaken with an equity lens, ensuring that regardless of socioeconomic status, all women and couples can use the method that meets their needs. This study explores the relationship between household wealth and the use of long-acting and permanent methods (LAPMs) versus short-acting methods of contraception among modern method users, using multivariate analyses based on Demographic Health Survey data from 30 developing countries conducted between 2006 and 2013. Overall, and controlling for relevant individual and household characteristics including age, number of living children, education, and urban/rural residence, we found that wealthier women were more likely than poorer women to use LAPMs instead of short-acting methods: 20 of the 30 countries showed a positive and statistically significant association between wealth and LAPM use. For 10 of those countries, however, LAPM use was significantly higher only for the top (1 or 2) wealthiest quintiles. Eight countries showed no broad pattern of association, while in 2 countries—Bangladesh and India—poorer women were more likely to use LAPMs than wealthier women. The positive association between wealth and LAPM use was found most consistently in the Latin American and the Caribbean countries in our sample. These findings can help program implementers respond better to women’s needs for modern contraception, especially in reaching women from lower- and middle-income households.

## Abstract (precis)

In general, across the developing world, wealthier women are more likely than poorer women to use long-acting and permanent methods of contraception instead of short-acting methods. Exceptions are Bangladesh, India, and possibly Haiti.

## INTRODUCTION

Although the Family Planning 2020 (FP2020) global movement has focused attention on improving access to modern contraception among the world’s poorest women, evidence suggests this goal is still far from reality.^1^ As programs continue to expand access to family planning information, services, and products, it is critical to undertake these efforts with an equity lens, ensuring that, regardless of socioeconomic status, all women and couples can use the method that meets their needs. In particular, for women and couples to make an informed choice, programs need to provide information about the benefits of long-acting and permanent methods (LAPMs), as well as access to those methods—either directly or through referrals. LAPMs comprise the long-acting and reversible methods of IUDs and implants as well as the permanent methods of tubal ligation and vasectomy. Benefits of LAPMs include convenience, effectiveness, cost-effectiveness, and potential health benefits,^2^^–^^4^ but overall use of LAPMs is still low in developing countries. Regional LAPM contraceptive prevalence rates average 4.2% and 21.9% in sub-Saharan Africa and Latin America, respectively.^5^

Many studies have demonstrated that wealth is positively associated with modern contraceptive use.^6^^-^^9^ However, it is unclear whether wealthier women are more likely than poor women to use LAPMs than short-acting methods. To our knowledge, only 3 studies have explored this issue.^7^^,^^10^^,^^11^ These studies suggest that wealth and LAPM use may be positively associated in developing countries. Creanga et al.^7^ conducted multivariate analysis of Demographic and Health Survey (DHS) data spanning 13 countries in sub-Saharan Africa and noted that use of long-acting contraceptive methods was more common among women in the wealthiest quintile than women in the poorest wealth quintile. However, by focusing only on the top and bottom wealth quintiles, that analysis left unanswered questions about access for the middle wealth quintiles. Using bivariate analysis, Ross and Agwanda^10^ explored the use of modern methods, in particular injectables, by wealth quintiles using data from DHS and the United Nations Development Programme (UNDP) in 28 countries—14 in Eastern and Southern Africa and 14 in West and Central Africa. The study found that women from wealthier households were more likely to be using pills, injectables, condoms, or female sterilization than women from poorer households. Although the results were informative, they do not provide insights into how household wealth is associated with use of one type of method over the others. Similarly, Fotso et al.^11^ analyzed DHS data from Kenya using multivariate regression and found that wealthier women were more likely to use LAPMs than poorer women, a disparity that increased from 2003 through 2008/2009.

Wealth is positively associated with modern contraceptive use, but the association between wealth and use of long-acting over short-acting methods is unclear.

Our analysis builds on these prior studies by conducting multivariate regression analysis in 30 countries in 3 regions to explore the relationship between household wealth and the type of contraceptive method used. Multivariate analysis allows us to correct for potential confounders (such as level of education or number of children) that are correlated with wealth and that may affect the choice between LAPMs and short-acting methods. To our knowledge, this is the first study to explore the relationship between wealth and type of method across all wealth quintiles, for many countries and different regions, using multivariate regression techniques to control for confounding factors.

Reasons that poor women may be less likely to use LAPMs could include barriers that programs need to address, such as financial costs, geographic barriers, medical and legal restrictions,^12^^-^^15^ provider bias and misinformation, social and cultural barriers,^16^ or simply different preferences. This paper cannot identify the reasons for non-use of LAPMs given data limitations. Rather, the purpose of this article is to determine whether a clear relationship exists between wealth and use of long-acting versus short-acting methods of contraception. Substantiating such a relationship allows the family planning community to advocate solutions to close this gap and find ways to remove barriers to LAPM use among poor women through formative and/or intervention research. Making this information available at the country level also helps countries understand whether this is a possible equity issue that needs to be resolved. However, it should be noted that proportions of women who are in need of LAPMs may be different by country because age structures and proportions of women wanting to limit childbearing may differ across countries.

## DATA AND METHODS

Our analysis used data from the DHS, which are household surveys that are nationally representative and internationally comparable, focusing primarily on reproductive health, fertility, and maternal and child health indicators. We used the most recent DHS data from 30 countries across 3 regions: 15 in sub-Saharan Africa; 9 in Asia and the Middle East; and 6 in Latin America and the Caribbean (Table 1). The surveys were conducted between 2006 and 2013.

**TABLE 1:** Countries and Survey Years Included in the Analysis

Region/Country	Survey Year
**sub-Saharan Africa**	
Burundi	2010
Cameroon	2011
Ethiopia	2011
Kenya	2009
Lesotho	2009
Madagascar	2008
Malawi	2010
Namibia	2006
Nigeria	2013
Rwanda	2010
Senegal	2012
Swaziland	2006
Tanzania	2010
Zambia	2007
Zimbabwe	2010
**Asia and the Middle East**	
Bangladesh	2011
Cambodia	2010
Egypt	2008
India	2006
Indonesia	2007
Jordan	2009
Nepal	2011
Pakistan	2007
Philippines	2008
**Latin America and the Caribbean**	
Bolivia	2008
Colombia	2010
Dominican Republic	2013
Guyana	2009
Haiti	2012
Peru	2008

Source: Demographic and Health Surveys.

Our final sample of countries was selected according to the following criteria. First, the most recent DHS was conducted in 2006 or later. Second, of the relevant sample of women of reproductive age (15–49 years) who were not currently pregnant and who had ever been sexually active, at least 10% reported currently using a modern contraceptive method. Third, the final sample for the country had to have at least 100 users of either LAPMs or short-acting methods among the relevant sample of women.

To assess the prevalence rates of traditional and modern contraception by type of method, we analyzed the sample of women of reproductive age who had ever been sexually active and who reported not being pregnant at the time of the interview. Table 2 displays the proportion of those women who reported using either no method, traditional methods, short-acting methods, or LAPMs for each country. Most countries in sub-Saharan Africa had low use of LAPMs, ranging from 1% in Cameroon and Nigeria to 7% in Kenya, Namibia, and Rwanda, and up to 11% in Malawi. Some countries in Asia and Latin America displayed much higher prevalence rates—from 27% in Jordan and Nepal to 39% in Egypt and the Dominican Republic to over 40% in India and Colombia.

Most sub-Saharan African countries have low LAPM use.

**TABLE 2:** Use of Contraception Among Women of Reproductive Age,^a^ by Type of Method

Country	None	Traditional	Short-acting	LAPMs	N
**sub-Saharan Africa**					
Burundi	78%	4%	14%	4%	5,660
Cameroon	69%	10%	20%	1%	11,940
Ethiopia	71%	1%	23%	4%	11,280
Kenya	58%	5%	29%	7%	6,414
Lesotho	56%	1%	39%	4%	6,156
Madagascar	60%	11%	26%	3%	13,872
Malawi	54%	4%	31%	11%	17,701
Namibia	41%	1%	50%	7%	7,679
Nigeria	80%	7%	12%	1%	25,244
Rwanda	55%	6%	33%	7%	8,591
Senegal	86%	1%	11%	2%	10,761
Swaziland	51%	2%	42%	5%	3,837
Tanzania	62%	7%	25%	6%	7,403
Zambia	61%	7%	30%	2%	5,440
Zimbabwe	44%	1%	50%	4%	6,765
**Asia and the Middle East**					
Bangladesh	39%	9%	44%	8%	16,654
Cambodia	51%	15%	28%	6%	11,912
Egypt	38%	3%	21%	39%	14,950
India	41%	8%	9%	43%	88,075
Indonesia	39%	4%	46%	11%	30,910
Jordan	36%	19%	18%	27%	8,851
Nepal	48%	7%	18%	27%	9,228
Pakistan	62%	10%	16%	12%	12,063
Philippines	50%	17%	21%	13%	8,889
**Latin America and the Caribbean**					
Bolivia	44%	23%	19%	13%	12,697
Colombia	28%	5%	26%	41%	42,242
Dominican Republic	31%	3%	26%	39%	7,524
Guyana	57%	3%	30%	11%	4,008
Haiti	69%	7%	21%	3%	7,861
Peru	36%	20%	30%	13%	31,261

Abbreviation: LAPMs, long-acting and permanent methods.

a Unit of analysis is women of reproductive (15–49 years old) who have ever been sexually active and who were not currently pregnant.

Source: Demographic and Health Surveys (various years).

For the multivariate analysis in this paper, we examined the subgroup of women who, in addition to being of reproductive age, sexually active, and not currently pregnant, also reported using a modern method of contraception (short-acting methods or LAPMs) at the time of the interview. In some countries in North Africa, the Middle East, and Asia (i.e., Bangladesh, Cambodia, Egypt, Indonesia, Jordan, Nepal, and Pakistan), the questions regarding access to and use of modern contraception were asked only to married women. Table 3 presents descriptive statistics for each study sample in the 30 countries: average age, average number of children alive at time of the interview, education, urban/rural residence, and distribution of the use of modern methods by type (LAPMs vs. short-acting methods). It also displays the numbers of observations of women who have all these requisites per survey that were considered for our analysis.

**TABLE 3:** Descriptive Statistics of Study Sample of Modern Contraceptive Users^a^

Country	Average Age	Average No. of Children	Education				Living in Urban Areas	Type of Modern Method Used		N
			None	Primary	High School	College or Higher		Short-Acting Methods	LAPMs	
**sub-Saharan Africa**										
Burundi	30.4	3.5	43%	44%	11%	2%	16%	78%	22%	1,075
Cameroon	27.3	2.0	4%	26%	58%	12%	73%	94%	6%	2,454
Ethiopia	29.5	3.1	51%	35%	7%	7%	33%	85%	15%	2,793
Kenya	31.7	3.1	3%	56%	30%	10%	30%	80%	20%	2,225
Lesotho	30.2	2.0	1%	43%	47%	9%	40%	91%	9%	2,489
Madagascar	30.7	3.2	12%	54%	32%	2%	19%	90%	10%	3,889
Malawi	30.5	3.5	15%	65%	18%	2%	21%	73%	27%	7,449
Namibia	29.7	2.0	4%	21%	66%	9%	59%	87%	13%	4,321
Nigeria	29.8	2.5	9%	20%	50%	22%	55%	90%	10%	3,117
Rwanda	31.5	3.3	16%	72%	10%	2%	14%	83%	17%	3,410
Senegal	31.9	3.5	45%	34%	18%	4%	69%	83%	17%	1,250
Swaziland	29.2	2.5	6%	28%	55%	12%	32%	89%	11%	1,837
Tanzania	30.5	3.1	14%	72%	14%	1%	33%	80%	20%	2,091
Zambia	29.5	3.3	10%	53%	30%	7%	46%	93%	7%	1,790
Zimbabwe	30.2	2.6	2%	30%	64%	5%	37%	92%	8%	3,690
**Asia and the Middle East**											
Bangladesh	29.8	2.4	25%	30%	36%	8%	27%	84%	16%	8,716
Cambodia	32.6	2.8	19%	57%	23%	1%	16%	83%	17%	3,993
Egypt	33.9	3.1	30%	12%	46%	12%	44%	35%	65%	8,524
India	34.2	2.9	45%	17%	32%	6%	35%	17%	83%	45,224
Indonesia	33.3	2.3	5%	48%	41%	7%	41%	81%	19%	16,963
Jordan	34.8	4.3	2%	5%	61%	32%	87%	40%	60%	3,831
Nepal	34.0	2.9	54%	18%	23%	5%	15%	40%	60%	4,194
Pakistan	34.4	4.2	51%	18%	21%	11%	41%	57%	43%	3,532
Philippines	33.7	3.1	0%	21%	46%	32%	53%	62%	38%	3,024
**Latin America and the Caribbean**										
Bolivia	32.0	2.8	4%	41%	34%	21%	73%	59%	41%	4,375
Colombia	32.7	2.1	2%	26%	49%	24%	79%	39%	61%	27,532
Dominican Rep.	33.1	2.5	2%	36%	36%	26%	75%	40%	60%	5,026
Guyana	32.1	2.5	1%	20%	70%	9%	32%	73%	27%	1,541
Haiti	29.7	2.5	21%	35%	38%	5%	50%	86%	14%	1,874
Peru	33.1	2.5	3%	26%	41%	30%	75%	70%	30%	13,770

Abbreviation: LAPMs, long-acting and permanent methods.

a Study sample is limited to women of reproductive age who have ever been sexually active, who were not pregnant at the time of the survey, and who reported current use of a modern contraceptive method.

Source: Demographic and Health Surveys (various years).

### Variables

Our outcome of interest was a dichotomous variable that was equal to *one* if the woman reported using a LAPM and *zero* if she reported using a short-acting method. Our main independent variable was household wealth, a variable that is included in the DHS datasets as a composite score, based on asset ownership and quality of housing; all surveyed households are ranked by index score and accordingly assigned to 1 of 5 wealth quintiles.^17^ This method of categorizing households based on wealth quintiles has been shown consistent with other wealth rankings, e.g., based on consumption expenditure aggregates, especially when other socioeconomic characteristics are taken into account.^18^^,^^19^ In the multivariate analysis, we represented this wealth variable as a vector of 5 dummy variables, with the poorest quintile (quintile 1) serving as the reference group.

Other control variables included:

- Age, in linear and quadratic form
- Number of living children, in linear and quadratic form
- Education, as a vector of dummy variables representing levels of education completed, including none (omitted category), elementary, secondary, and tertiary education
- A vector of dummy variables for employment status and type of occupation of the head of the household (the omitted category was for households in which the head either was unemployed or performed manual or domestic tasks for a living)
- A dummy variable for marital status equal to one for women who were married or living in union, and zero otherwise (this variable was used as a control only for those countries where questions regarding use of contraception were asked regardless of marital status, as previously explained)
- Urban/rural residence

We included this vector of controls in order to isolate more precisely the relationship between use of LAPMs versus short-acting methods and wealth, as a proxy for disposable income. Age and education, for example, are variables that are correlated with both wealth and use of either type of method. Controlling for them is equivalent to exploring the nature of the relationship between wealth and use of LAPMs within women of the same age or same level of education. Although some of these controls are highly correlated, in order to improve the precision of our estimates and reduce the potential for omitted variable bias, all of them were included as controls simultaneously.

We hypothesized that most countries would show a positive association between wealth and use of LAPMs (versus short-acting methods). It was expected that this could occur in the form of a positive linear relationship across all wealth quintiles (meaning that women from higher quintiles would always be more likely to use LAPMs than women from lower quintiles) or in the form of a non-linear relationship, i.e., just for the highest quintiles (meaning that women from only the top 1 or 2 highest quintiles would be more likely to use LAPMs, but women from lower quintiles would be equally likely to use LAPMs than women from the lowest quintile). A negative relationship would mean that wealthier women would be less likely to use LAPMs than poorer women.

### Analytical Methods

First, we examined the unadjusted relationship between our outcome of interest (use of LAPMs versus short-acting methods) and our main independent variable (wealth) separately for the 30 countries in our final sample. Second, we ran (adjusted) multivariate logistic regressions for each country, in order to control for specific individual and household characteristics that can confound the relationship between wealth and contraceptive method of choice. These control variables were age, number of living children, educational attainment, employment status and type of occupation of head of household, marital status (when applicable), and urban/rural residence.

The multivariate analysis was performed using logistic regression models to yield coefficient estimates displayed as odds ratios (ORs). These ratios represent the odds of an individual using a LAPM over the odds of using a short-acting method (thus, an odds ratio larger than one implies that the individual is more likely to use a LAPM than a short-acting method). We used the within-country weighting variables specified by each country-specific DHS. Occasionally, a country presented strata with a single sampling unit in our regressions; those strata were treated as certainty sampling units.^20^

## RESULTS

### Unadjusted Analysis

The unadjusted relationship between wealth and use of LAPMs (versus short-acting methods) among modern method users varied across countries (Table 4). The complement of each proportion reported in Table 4 is, by definition, the proportion of women using short-acting methods. In Burundi, for example, among the sampled women in the poorest quintile (quintile 1), 22% used LAPMs and the remaining 78% used short-acting methods.

**TABLE 4:** Proportion of Modern Method Users^a^ Using LAPMs, by Wealth Quintile

Country	Wealth Quintile				
	Q1 (Poorest)	Q2	Q3	Q4	Q5 (Wealthiest)
**sub-Saharan Africa**					
Burundi	22%	15%	20%	23%	26%
Cameroon	7%	8%	8%	7%	5%
Ethiopia	16%	21%	16%	16%	12%
Kenya	14%	16%	21%	21%	21%
Lesotho	3%	6%	7%	10%	11%
Madagascar	8%	7%	7%	12%	13%
Malawi	24%	23%	26%	28%	32%
Namibia	5%	6%	8%	13%	22%
Nigeria	6%	8%	10%	9%	12%
Rwanda	12%	15%	15%	18%	25%
Senegal	21%	17%	14%	15%	19%
Swaziland	6%	9%	10%	9%	16%
Tanzania	17%	24%	20%	22%	18%
Zambia	2%	5%	6%	5%	13%
Zimbabwe	6%	6%	5%	7%	14%
**Asia and the Middle East**					
Bangladesh	21%	18%	16%	13%	10%
Cambodia	14%	14%	12%	17%	31%
Egypt	53%	61%	64%	72%	73%
India	90%	89%	88%	83%	71%
Indonesia	14%	17%	17%	19%	29%
Jordan	56%	59%	57%	60%	69%
Nepal	54%	64%	66%	64%	50%
Pakistan	50%	45%	47%	39%	40%
Philippines	33%	38%	39%	40%	41%
**Latin America and the Caribbean**						
Bolivia	25%	33%	39%	44%	51%
Colombia	61%	62%	61%	62%	59%
Dominican Republic	52%	58%	65%	62%	62%
Guyana	24%	27%	29%	27%	26%
Haiti	25%	18%	19%	11%	10%
Peru	18%	26%	27%	34%	34%

Abbreviations: LAPMs, long-acting and permanent methods; Q, quintile.

a Study sample is limited to women of reproductive age who have ever been sexually active, who were not pregnant at the time of the survey, and who reported current use of a modern contraceptive method.

Source: Demographic and Health Surveys (various years).

Overall, in 17 of the 30 countries, a greater proportion of women in the wealthiest quintile used LAPMs compared with women in the poorest quintile. Conversely, of course, short-acting methods were used by a greater proportion of poorer women than wealthier women. **This positive (and linear) relationship between wealth and use of LAPMs was the dominant pattern in each region**, observed in half to two-thirds of the countries sampled: 10 of the 15 African countries, 5 of the 9 Asian/Middle Eastern countries, and 3 of the 6 Latin American/Caribbean countries.

In most countries, wealthier women were more likely to use LAPMs than poorer women.

There were many exceptions to this pattern, however. Four countries—Bangladesh, Haiti, India, and Pakistan—exhibited a clearly negative relationship: LAPM use was far more common among poorer women than among wealthier women. For another 4 countries (the Dominican Republic, Ethiopia, Nepal, and Tanzania), the relationship resembled an inverted U-shape, with LAPM use higher in the middle wealth quintiles and lower in both the poorest and the wealthiest quintiles. In Burundi and Senegal, the relationship resembled a U-shape, with LAPM use more likely in both the poorest and the wealthiest quintiles and lower in the middle quintiles; however, no large differences were observed in the proportions across all quintiles. Finally, in Cameroon, Colombia, and Guyana, there appeared to be no relationship between wealth and type of method used.

### Multivariate Analysis

We used multivariate logistic regression to control for a vector of potentially confounding covariates: age, number of living children, education, employment/occupation, marital status, and urban/rural residence. Table 5 shows the adjusted odds ratios from the multivariate regressions, organized by region.

**TABLE 5:** Adjusted Odds Ratios for Relationship Between Wealth Quintile and Use of LAPMs vs. Short-Acting Methods Among Study Sample of Modern Method Users^a^

Country	Q1 (Poorest)	Q2	Q3	Q4	Q5 (Wealthiest)	N
**sub-Saharan Africa**						
Burundi	1.00	0.59	0.81	0.98	0.99	1,075
Cameroon	1.00	2.04	2.13	2.69	2.70	2,454
Ethiopia	1.00	1.47	1.07	1.04	0.81	2,793
Kenya	1.00	0.96	1.21	1.35	2.61**	2,225
Lesotho	1.00	2.53*	2.46+	5.24**	6.59**	2,489
Madagascar	1.00	0.79	0.70	1.31	1.21	3,889
Malawi	1.00	0.99	1.21	1.30+	1.74**	7,449
Namibia	1.00	1.02	1.25	2.37**	5.08**	4,318
Nigeria	1.00	1.65	2.17*	2.27*	2.28*	3,117
Rwanda	1.00	1.22	1.38+	1.57*	2.02**	3,410
Senegal	1.00	0.74	0.48*	0.56	0.83	1,250
Swaziland	1.00	1.55	1.63	1.34	1.86	1,837
Tanzania	1.00	1.62+	1.37	1.94*	1.48	2,091
Zambia	1.00	3.31*	4.51**	4.71**	11.34**	1,790
Zimbabwe	1.00	1.04	0.72	1.05	1.80+	3,690
**Asia and the Middle East**						
Bangladesh	1.00	0.80*	0.69**	0.59**	0.54**	8,755
Cambodia	1.00	0.96	0.78	0.99	1.76*	3,993
Egypt	1.00	1.29**	1.42**	1.97**	1.94**	8,524
India	1.00	0.87*	0.83**	0.77**	0.53**	45,224
Indonesia	1.00	1.15	1.05	1.04	1.41*	16,963
Jordan	1.00	1.09	1.04	1.12	1.58+	3,831
Nepal	1.00	1.74**	1.97**	2.33**	1.84**	4,194
Pakistan	1.00	0.85	1.05	0.88	1.06	3,532
Philippines	1.00	1.18	1.05	1.08	1.20	3,024
**Latin America and the Caribbean**						
Bolivia	1.00	1.80**	2.23**	2.72**	3.19**	4,375
Colombia	1.00	1.34**	1.45**	1.70**	1.68**	27,532
Dominican Republic	1.00	1.52*	1.74**	1.40+	1.84**	5,026
Guyana	1.00	1.27	1.50	1.50	1.53	1,541
Haiti	1.00	0.55+	0.58	0.52	0.54	1,874
Peru	1.00	1.42*	1.72**	2.65**	2.23**	13,770

Abbreviations: LAPMs, long-acting and permanent methods; Q, quintile.

a Study sample is limited to women of reproductive age who have ever been sexually active, who were not pregnant at the time of the survey, and who reported current use of a modern contraceptive method.

+ *P* < .10,

* *P* < .05,

** *P* < .01

Source: Demographic and Health Surveys (various years).

The 30 countries exhibited 1 of 4 patterns:

- A consistently positive, statistically significant relationship across all 5 wealth quintiles, such that wealthier women were more likely to use LAPMs and women in the lowest wealth quintiles were more likely to use short-acting methods.
- A positive association between wealth and LAPM use, *only* when comparing the top 1 or 2 wealthiest quintiles with the poorest quintile, and no significant difference in LAPM use between the lower wealth quintiles and the poorest quintile. That is, women from the lowest 2 or 3 quintiles showed no systematic preference for LAPMs or short-acting methods, and the wealth effect was apparent only in the highest wealth quintiles.
- No significant differences in LAPM use, or a significant difference only when comparing the lower or middle quintiles with the poorest quintile. In these countries, wealth did not appear to be associated positively or negatively with LAPM use.
- A significant negative association between wealth and LAPM use, such that wealthier women were more likely to use short-acting methods and poorer women were more likely to use LAPMs.

These patterns varied by region. In **sub-Saharan Africa**, as noted in the unadjusted analysis, 10 of the 15 countries analyzed showed statistically significant and positive relationships overall between wealth and LAPM use. In 3 of these 10 countries (Lesotho, Nigeria, and Zambia), women from almost all upper quintiles were significantly more likely to use LAPMs than women in the poorest quintile. In 6 of the 10 countries (Kenya, Malawi, Namibia, Rwanda, Swaziland, and Zimbabwe), a significant relationship was found only when comparing the top 1 or 2 wealth quintiles with the poorest quintile. Tanzania showed a significant positive association only when comparing the second and fourth quintiles. In the other 5 sampled sub-Saharan African countries (Burundi, Cameroon, Ethiopia, Madagascar, and Senegal), no statistically significant relationship was found when comparing the poorest quintile with any other quintile.

In **Asia and the Middle East**, a positive and statistically significant relationship between wealth and LAPM use was noted in 5 countries: Cambodia, Egypt, Indonesia, Jordan, and Nepal. In 3 of those countries—Cambodia, Indonesia, and Jordan—the relationship was significant only for women in the wealthiest quintile. In 2 other countries—Bangladesh and India—a significant *negative* relationship was found: in those countries, as discussed below, poorer women were more likely than wealthier women to use LAPMs rather than short-acting methods. No association was found for Pakistan or the Philippines.

In Bangladesh and India (and possibly Haiti), poorer women were more likely than wealthier women to use LAPMs than short-acting methods.

Finally, in **Latin America and the Caribbean**, 5 of the 6 countries showed positive and statistically significant associations between wealth and LAPM use, mostly across all wealth quintiles. Haiti was the exception—although the association was not statistically significant, the average odds ratio was around 0.54, suggesting that women from the lowest quintile may be more likely than wealthier women to use LAPMs than short-acting methods, as in Bangladesh and India

There was wide variation in the magnitude of the outcome differences. Some countries showed a markedly larger likelihood of LAPM use for women from the wealthiest quintile. Four countries—Bolivia, Lesotho, Namibia, and Zambia—had odds ratios greater than 3 when comparing outcomes between the wealthiest quintile and the poorest quintile, showing the strongest (adjusted) association between wealth and LAPM use.

## DISCUSSION

Using recent data from 30 countries in 3 regions, we examined patterns of use of LAPMs and short-acting methods in relation to household wealth. Our analyses showed a general pattern of greater LAPM use by wealthier women: 20 of the 30 countries showed some pattern of positive and statistically significant association between wealth and LAPM use. However, for 10 of those 20 countries, this pattern was limited to a comparison between the wealthiest 1 or 2 quintiles and the poorest quintile; there was no significant difference between usage by women from the poorest households and women from middle-income households. **These findings suggest that in many countries the income threshold is high**—for reasons that remain to be explored.

The remaining 10 countries analyzed demonstrated 2 different patterns. No significant relationship was found between wealth and type of method used in 8 countries: Burundi, Cameroon, Ethiopia, Madagascar, and Senegal; Pakistan and the Philippines; and Haiti. The other pattern was a significant inverse (negative) relationship between wealth and LAPM use in Bangladesh and India: poorer women were more likely to use LAPMs than wealthier women, and wealthier women were more likely to use short-acting methods than poorer women. This inverse pattern may reflect a different policy environment in these countries, where supply-side and demand-side incentives, reinforced by community mobilization, contribute to high uptake of LAPMs among the poor. In Bangladesh, for example, LAPM service delivery has been prioritized by the government and is backed with a large budget, including funds for client compensation and provider fees.^21^ In India, female sterilization is the leading method of contraception, accounting for two-thirds of all current contraceptive use and about three-quarters of all modern method use^22^; it is provided free of charge in the public sector^23^ and has a long history of government promotion as the primary method of family planning.^24^ Although the Indian government ceased to announce national sterilization targets in 1996, there is some evidence that targets and reimbursements to cover costs such as travel expenses are still used to encourage female sterilization.^25^^,^^26^

**Figure:**
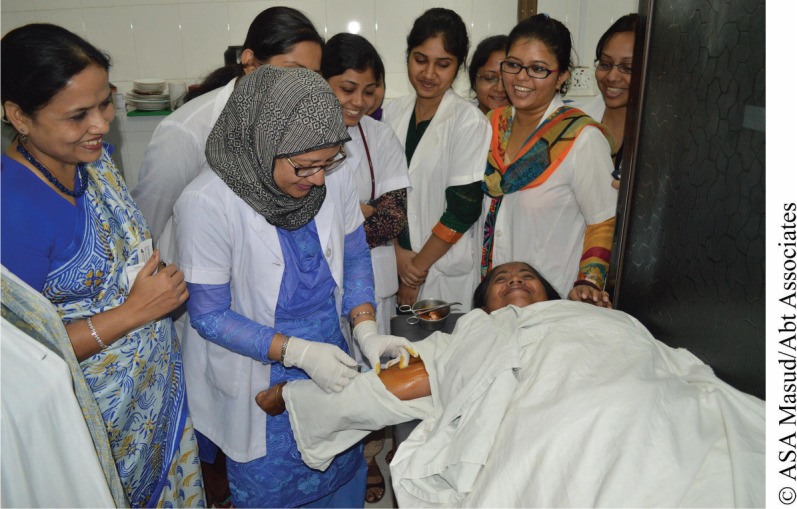
A medical intern in Bangladesh inserts a contraceptive implant in a client’s arm under supervision while other interns watch and learn.

While our study did not analyze use of specific LAPMs (e.g., IUDs vs. female sterilization) by wealth, we can assume the patterns are generally similar to regional patterns among all contraceptive users. For example, in Latin America and the Caribbean, out of a modern contraceptive prevalence rate (mCPR) of 58.1%, almost one-third (31.4%) is from LAPMs, with a strong presence of female sterilization and, to a lesser extent, IUDs.^27^ In countries from Asia, the Middle East, and North Africa, where the mCPR is 51.7%, LAPMs contribute 14.3% to that mCPR, with 8% of women using IUDs and approximately 5% using sterilization. In countries from sub-Saharan Africa, mCPR is 26.5% and overall LAPM use is low at less than 5%, with less than 2% of women using implants and less than 2% using sterilization.

The positive relationship between wealth and LAPM use is an issue of concern as it may indicate that there is inequity in access to LAPMs in many developing countries. These differentials may be due to several reasons and have different remedies. Poor women may be less likely to use LAPMs due to financial barriers, which could be addressed by voucher programs that subsidize the costs of LAPM services. Similarly, contracting-out through NGOs could improve access to these methods so that women do not have to pay full price through private providers and facilities. Geographic barriers may be an issue for poor women, which is more difficult to address. This may require more concerted efforts to provide LAPMs through high-quality, mobile outreach services in poor areas.^28^ In addition, the expansion of social franchising programs can remove geographic barriers by training providers in hard-to-reach areas in the provision of LAPMs, while ensuring they have needed supplies and quality standards.^29^^,^^30^ Lack of information among women and/or their spouses may lead couples to be less likely to use LAPMs. Addressing this issue would require a concerted effort by both the public and the private sectors, so that messages focus on the benefits of these methods rather than on the specific type of provider. For example, in Jordan, a private-sector health project funded by the United States Agency for International Development (USAID) supported a behavior change communication campaign that focused on the benefits of using IUDs. The project demonstrated changes in knowledge, attitudes, and intention to use IUDs without focusing on the source of the method (public versus private sector).^31^ Addressing the problem of lack of information about LAPMs also requires that community health workers who do not provide the methods be conversant in the benefits and referral systems so that women and couples can access the full range of methods that helps them achieve their reproductive goals. It also may be possible that poorer women simply have different preferences. Follow-up formative research and intervention testing is required to disentangle the reasons we find this strong association across a large number of countries.

Inequity in access to LAPMs may be an issue in many developing countries.

### Limitations

The analysis has several limitations. First, the DHS data do not provide specifics such as location and proximity to services, which influence access to methods. These characteristics may be correlated with both household wealth and contraceptive choice. Second, for the purposes of this analysis we have grouped all types of LAPMs together due to the issue of sample size; however, we might find differing patterns for long-acting *reversible* methods (IUDs and implants) versus *permanent* methods (male and female sterilization). Third, in a few countries, the use of any modern method was low across all wealth quintiles, but especially in the lowest wealth quintiles; in those countries (Nigeria is a good example), we found no statistical significance across the key wealth coefficients, but that may be due to small sample size of women from those quintiles using modern methods. Fourth, this analysis provides a snapshot of current behavior; it does not capture change over time. Despite its limitations, this paper demonstrates a strong, positive relationship between wealth and LAPM use in many developing countries that deserves further exploration.

## CONCLUSION

In most developing countries, wealthier women are more likely than poorer women to use long-acting and permanent methods of contraception than short-acting methods. Notable exceptions are Bangladesh, India, and possibly Haiti, where poorer women are more likely to use long-acting and permanent methods than wealthier women, perhaps reflecting a different policy environment than in other countries.

## Notes

**Peer Reviewed**

**First Published Online:** 2016 Feb 17
